# Harming by Deceit: Epistemic Malevolence and Organizational Wrongdoing

**DOI:** 10.1007/s10551-023-05370-8

**Published:** 2023-02-24

**Authors:** Marco Meyer, Chun Wei Choo

**Affiliations:** 1 Faculty of Philosophy, University of Hamburg, University of Hamburg, Überseering 35, Postfach #4, 22297 Hamburg, Germany; 2 Faculty of Information, University of Toronto, 140 St. George Street, Toronto, ON M5S 3G6 Canada

**Keywords:** Epistemic vice, Organizational wrongdoing, Epistemic malevolence, Deception, Organizational behavior

## Abstract

Research on organizational epistemic vice alleges that some organizations are epistemically malevolent, i.e. they habitually harm others by deceiving them. Yet, there is a lack of empirical research on epistemic malevolence. We connect the discussion of epistemic malevolence to the empirical literature on organizational deception. The existing empirical literature does not pay sufficient attention to the impact of an organization’s ability to control compromising information on its deception strategy. We address this gap by studying eighty high-penalty corporate misconduct cases between 2000 and 2020 in the United States. We find that organizations use two different strategies to deceive: Organizations ‘sow doubt’ when they contest information about them or their impacts that others have access to. By contrast, organizations ‘exploit trust’ when they deceive others by obfuscating, concealing, or falsifying information that they themselves control. While previous research has focused on cases of ‘sowing doubt’, we find that organizations ‘exploit trust’ in the majority of cases that we studied. This has important policy implications because the strategy of ‘exploiting trust’ calls for a different response from regulators and organizations than the strategy of ‘sowing doubt’.

## Introduction

Organizations can do great harm by deceiving customers, stakeholders, and society more broadly. For instance, scientists working at tobacco companies had observed that smoking causes cancer in their own clinical experiments as early as in the 1950s (Derry & Waikar, 2008, p. 111). Instead of sharing these findings with the public, tobacco companies created the Tobacco Industry Research Committee to conceal the harmful effects of smoking, discrediting serious research and promoting junk science. The car manufacturer Volkswagen installed ‘defeat devices’ in its diesel engines to change performance to meet emissions standards only when tested by regulators (Katz, 2017). Amazon copied successful products and manipulated search results on its marketplace to promote its products over those by third-party vendors (Kalra & Stecklow, 2021).

These examples are cases of ‘epistemic malevolence.’ Baird and Calvard (2019) describe epistemic malevolence as one type of epistemic vice prevalent in organizations. Epistemic vices are dispositions, patterns of behavior, and attitudes that undermine knowledge. These features are rooted in the organization’s culture and governance (Battaly, 2017; Cassam, 2019). Epistemic malevolence is one such epistemic vice, i.e. the disposition to harm others by deceiving them (Baehr, 2010; Cassam, 2018).

Organizations use deceit frequently to cover up material harms they caused (Fleming & Zyglidopoulos, 2008; Jenkins & Delbridge, 2020). For instance, BP initially lied about the amount of oil that it had spilled in the Gulf of Mexico during the Deepwater Horizon explosion of 2010 (Beyer et al., 2016). By contrast, epistemic malevolence is about organizational deceit that leads others to make bad decisions which in turn cause material harm. For instance, obscuring knowledge about the health effects of smoking leads to fewer people quitting smoking, and manipulating emission tests leads customers to buy cars they would not otherwise buy. Other material harms caused by companies we study relate to the financial, the educational, and physical well-being of customers and other stakeholders.

The purpose of this paper is to explore the role of epistemic malevolence in organizational wrongdoing. Previous research on organizational epistemic vice (Baird & Calvard, 2019; Bland, 2022; Fricker, 2010; Lamy, 2022; Rawwas et al., 2013; Rooij & Bruin, 2022) has not investigated epistemic malevolence in depth. There is also a lack of empirical research on epistemic malevolence in relation to organizational wrongdoing. We connect the discussion of epistemic malevolence to the empirical literature on organizational deception. An area that existing empirical literature on organizational deception does not shed much light on is the impact of an organization’s ability to control compromising information on its deception strategy.

We address this gap by studying eighty high-penalty corporate misconduct cases between 2000 and 2020 in the United States. We find evidence of epistemic malevolence in 60% of the cases that we study. Depending on their level of control over compromising information, we distinguish two different strategies that organizations use to deceive: Organizations ‘sow doubt’ when they contest information about them or their impacts that others control. Organizations ‘exploit trust’ when they deceive others by obfuscating, concealing, or falsifying information that they themselves control. Previous research has focused on cases of ‘sowing doubt’. For instance, Michaels (2020, ch. 1 and 7) describes how pharma companies contributed to the opioid epidemic using this strategy, for instance by sowing confusion to buy time and mobilizing resources to discredit data. Sowing doubt is also the strategy behind the research committee set up by tobacco companies to promote junk science mentioned above (Derry & Waikar, 2008; Michaels, 2008; Michaels & Monforton, 2005; Oreskes & Conway, 2011).

By contrast, we find that organizations ‘exploit trust’ in the majority of cases that we studied (Harris & Zaheer, 2006; Zaheer et al., 1998). We draw attention to the ways in which epistemically malevolent organizations create an appearance of trustworthiness and exploit this appearance to betray external stakeholders. Unlike what the paradigm of ‘sowing doubt’ would suggest, organizations are not merely trying to contest findings by researchers in these cases. Rather, they preempt researchers, regulators, journalists, and NGOs from investigating the harms they cause in the first place. Sometimes, organizations even set up sophisticated systems that allow them to exercise exclusive control over the flow and interpretation of information, as in the cases of Volkswagen and Amazon mentioned above. This has important policy implications because the strategy of ‘exploiting trust’ calls for a different response from regulators and organizations than the strategy of ‘sowing doubt’.

The paper is organized as follows: We first introduce the notion of epistemic malevolence as a collective epistemic vice, situating it in the discussion in virtue and vice epistemology. We then connect the philosophical literature to the empirical research on organizational deception and introduce the distinction between the strategies of ‘sowing doubt’ and ‘exploiting trust’ by drawing on the notion of information use environments developed in information science (Taylor, 1991). This sets us up for presenting our empirical study. After introducing the data and methodology, we present the findings, followed by a general discussion and a discussion of limitations and opportunities for future research.

### Epistemic Malevolence as an Epistemic Vice of Organizations

In line with the virtue-responsibilist strand in virtue epistemology (Zagzebski, 1996), we conceive of epistemic vices as trait-like dispositions that interfere with gaining, keeping, or sharing knowledge (Cassam, 2019; Crerar, 2018; Tanesini, 2018), such as close-mindedness, intellectual arrogance, and prejudice. We set aside the virtue-reliabilist conception of epistemic vice as deficient cognitive faculties, such as memory or perception (Sosa, 1985). One difference between the two conceptions is that for the virtue-responsibilist, but not for the virtue-reliabilist, epistemic vices differ from cognitive defects. The reason is that virtue-responsibilists insist that, unlike cognitive defects, epistemic vices are reprehensible because their bearers are responsible either for acquiring these vices or for continuing to display them (Cassam, 2019, p. 20ff). Epistemic vices form the mirror-image of epistemic virtues, which are traits that support the gaining, keeping, and sharing of knowledge (Montmarquet, 1993; Roberts & Wood, 2007; Zagzebski, 1996). Individuals who exhibit higher levels of epistemic vice are more likely to believe conspiracy theories, find fake news credible, and buy into myths about Covid-19 (Meyer et al., 2021).

Researchers in business ethics and business studies have fruitfully applied the notion of epistemic virtue and vice to organizations, exploring their importance in an organizational context (Alzola, 2008; Rawwas et al., 2013), using the concept to describe a new approach to business ethics (Arjoon, 2000; de Bruin, 2013) and to systematically lay out the ways in which organizations may deal poorly with information (Lamy, 2022).

Yet whether epistemic vices can truly be possessed by organizations has been subject to considerable discussion (de Ridder, 2022; Fricker, 2010; Lahroodi, 2007). De Ridder (2022) has systematized three ways in which epistemic virtues and vices can be attributed to collectives such as organizations. First, collectives can be epistemically vicious in an ‘additive fashion’ if all or most of its members are epistemically vicious (de Bruin, 2014). Second, ‘interaction’ due to the collective’s governing structure or culture can generate a disposition to epistemically vicious behavior (Dempsey, 2015; Miller, 2010). Third, collectives can display ‘emergent’ collective epistemic virtues and vices, i.e. virtues and vices that only collectives can possess, such as solidarity (Battaly, 2022). Moreover, epistemic virtues and vices have been used to explore the behavior of organizations (de Bruin, 2014; Moore, 2005; Moore & Beadle, 2006). Baird and Calvard (2019) have conceptually distinguished four epistemic vices as particularly pertinent for understanding organizational wrongdoing: Epistemic malevolence, epistemic insouciance, epistemic hubris, and epistemic injustice.

In what follows, we focus on the role of epistemic malevolence in organizational wrongdoing. Existing research on organizational epistemic vice has mostly focused on epistemic vices that primarily undermine the knowledge of the vicious organization itself, such as closed-mindedness or indifference (de Bruin, 2014). By focussing on epistemic malevolence, we explore how organizations undermine the knowledge of others (Baehr, 2010, p. 204; Cassam, 2018, p. 13). We explore the extent to which wrongdoing is rooted in an organization’s culture and governance, and describe the information strategies epistemically malevolent organizations adopt.

The *vice* of epistemic malevolence differs from a mere *behavior* in that the vice is a disposition. To exhibit epistemic malevolence, it is therefore not enough to merely deceive or mislead another person on one occasion. Rather, this behavior must be grounded in a motivation to deceive others when it serves your interest (Baehr, 2010). Isolated episodes of epistemically malevolent behavior may be due to adverse circumstances rather than epistemic vice, with people or organizations acting “out of character” (Cassam, 2018, p. 18). Hence, isolated episodes of epistemically malevolent behavior are insufficient to attribute the vice of epistemic malevolence to an organization. However, having an epistemic vice can *explain* that their bearers deceive or mislead because the vice of epistemic malevolence gives rise to epistemically malevolent behaviors in suitable circumstances (Cassam, 2016).

In this article, we attribute epistemic virtues and vices and deceptive behavior to organizations. This approach runs the risk of personifying organizations.^1^ Jensen and Meckling warn against “ thinking about organizations as if they were persons with motivations and intentions” (Jensen & Meckling, 2000, p. 311). We attribute behavior to organizations as a shorthand to characterize behaviors of organization members shaped by the cultural environment of the organization and its governance structures (Chen et al., 1997; Dempsey, 2015; Kaptein, 2011; Mejia & Skorburg, 2022). We do not imply that organizations are homogenous entities, or take a stance on how blame should be apportioned between organizations and their members.

### Conceptualizing Epistemic Malevolence as a Type of Deception

Epistemic malevolence has primarily been discussed in the field of social epistemology. As a result, there is an emphasis on theoretical inquiry, conducted in the vernacular of philosophy. There is however extensive empirical research on deception that addresses epistemic dimensions (Buller et al., 1994; Burgoon et al., 1996; Hubbell, 2019). The purpose of this section is to connect the literature on epistemic malevolence to research on organizational deception. Making this connection has two benefits. First, it enriches the philosophical discussion on epistemic malevolence with empirical insights. Second, we hope to draw the attention of researchers in organization studies to epistemic malevolence as a particular type of organizational deception that needs to be addressed in novel ways, as we argue below.

We propose to conceptualize organizational epistemic malevolence as a particular type of organizational deception: deception that leads to material harm due to decisions of the deceived, and that is rooted in features of the organization that are stable to a certain extent, such as culture and governance. We define organizational culture as a pattern of shared basic assumptions that shapes members’ way to perceive, think, and feel in relation to organizational problems (Schein, 2010). We define governance as the system by which an organization is controlled and operated, and by which its members are held to account (Mallin, 2016). Our proposed definition of epistemic malevolence cannot do justice to some of the nuances of the philosophical debate. But it has the advantage of conceptualizing epistemic malevolence in a way that has been studied empirically, and will provide the ground for our own empirical analysis.

Organizational deception has been studied empirically from a range of perspectives, including studies of the individual (Gaspar et al., 2021; Helzer et al., 2022), behavioral (Grover, 1993; Leavitt & Sluss, 2015; Shalvi et al., 2011; Xie et al., 2022) and contextual antecedents of deceptive behavior (Olekalns et al., 2014; Sims, 2002), as well as the mechanisms that can lead to the escalation and festering of deception in organizations (Fleming & Zyglidopoulos, 2008; Jenkins & Delbridge, 2017). There has also been work elucidating the ethics of deception in marketing (Sher, 2011), policing (Alpert & Noble, 2009), business negotiations (Sherwood, 2021), and sales (Carson, 2001).

Based on a comprehensive review of the literature, Masip et al. (2004) developed a general definition of deception: “Deception is defined as the deliberate attempt, whether successful or not, to conceal, fabricate, and/or manipulate in any other way factual and/or emotional information, by verbal and/or nonverbal means, to create or maintain in another or in others a belief that the communicator himself or herself considers false” (Masip et al., 2004, p. 147f.). Hence, deception has three features: First, it is intentional. The empirical literature often studies it in the framework of strategic interaction (Hubbell, 2019). This feature is shared by epistemic malevolence. Second, it involves the manipulation of information. Buller et al. (1994) and Burgoon et al. (1996) distinguish among three general modes of manipulating information: concealment that withholds or omits information; equivocation that presents information ambiguously; falsification that presents information which the deceiver knows to be false. Note that all of these behaviors are epistemic, establishing a further point of contact between the concepts of epistemic malevolence and of deception. Third, deception aims at creating or maintaining a false belief in others. We set aside self-deception, which does occur in organizations and has been studied as an enabler of unethical decision-making (Caldwell, 2009; Tenbrunsel & Messick, 2004). In line with the other-regarding focus of epistemic malevolence, our focus here on deception aimed at others.

Despite these similarities between deception and epistemic malevolence, they are not identical. First, epistemic malevolence concerns only ‘malevolent’ deception. Jenkins and Delbridge (2020) distinguish four purposes of organizational lies, which apply to deception more generally: principled, defensive, malicious, and material. Principled and defensive deceptions are benevolent: Principled deception protects others from harm, and defensive deception aims at protecting the interests of others. By contrast, malicious and material deception are malevolent.

Second, epistemic malevolence concerns only deception that leads to harm due to decisions of the deceived. In their study of how deception escalates in organizations, Fleming and Zyglidopoulos (2008) distinguish between what they call “deception for its own sake” and deception that “supports other forms of wrongdoing” (p. 838). We propose a related distinction between deception that leads the deceived to make decisions causing material harm; and deception that merely ‘covers up’ material harm independently caused by the organization. Epistemic malevolence concerns only the former type of deception: cases where the decisions of the deceived cause material harm.

Third, while the deception literature focuses on behavior, epistemic malevolence concerns deceptive behavior only to the extent that it is rooted in a stable disposition. In individuals, virtues and vices are rooted in people’s character (Battaly, 2015; Jayawickreme & Fleeson, 2017). To apply the idea of vices to organizations, we need a functional equivalent of character that grounds a disposition. An organization’s culture and governance are good candidates for grounding dispositions (Aikin & Clanton, 2010; Dempsey, 2015; Fricker, 2010; Mejia & Skorburg, 2022; Trevino, 1986). Ermann and Lundman (2002) argue that the governance and culture of organizations can encourage wrongdoing in at least three ways: by limiting information and responsibility; by establishing norms, rewards, and sanctions that encourage deviance; and through organizational elites who initiate deviance and use their hierarchical positions to implement it. In organization studies and sociology, there is a related discussion about the impact of context on organizational behavior (Johns, 2001, 2006).^2^ Johns maintains that situational opportunities and constraints affect organizational behavior. As a result, research on organizational behavior is best conducted across multiple levels of analysis, focusing on (1) individual factors, (2) institutional factors, e.g. the level of teams or the organization as a whole, and (3) structural factors external to the organization (Jepperson & Meyer, 2011).

**Fig. 1:**
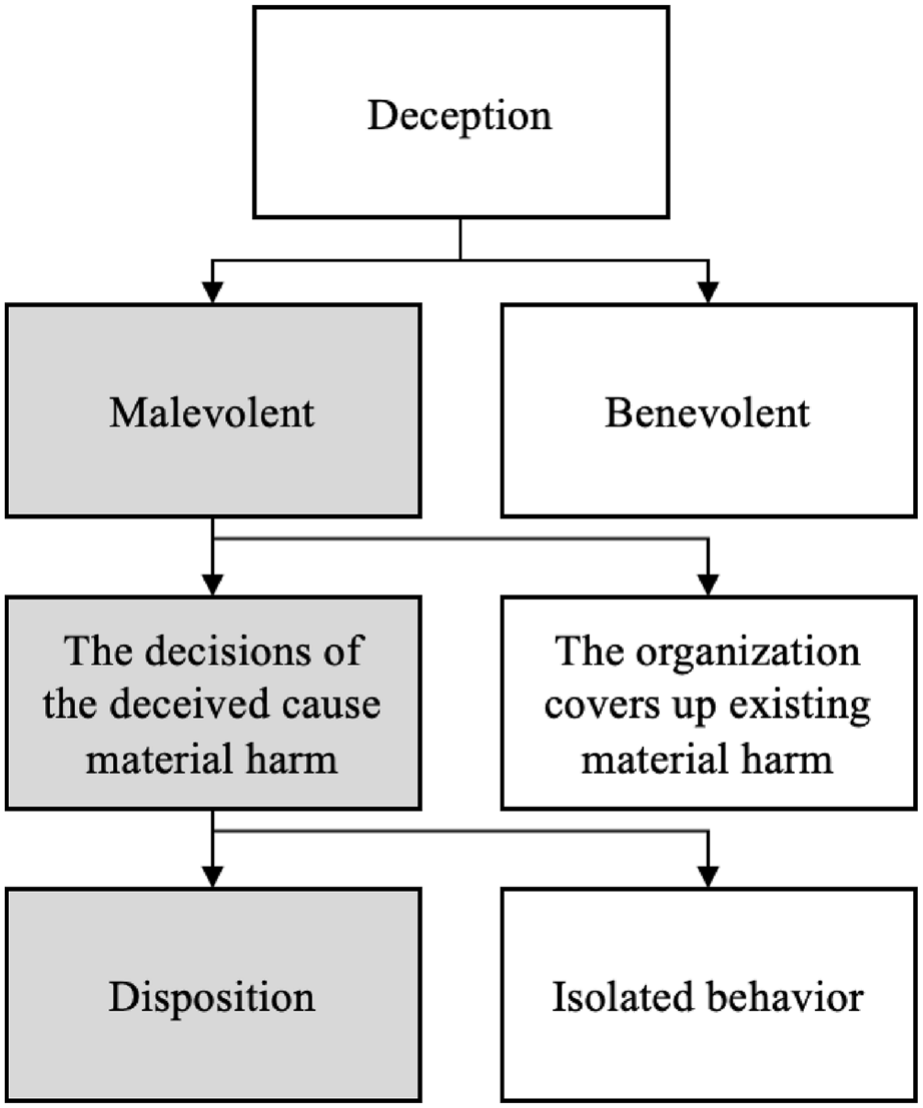
Organizational epistemic malevolence is a disposition to deceive that is malevolent and constitutes harm (options in gray capture features of epistemic malevolence)

Figure 1 summarizes the relationship between deception and epistemic malevolence, characterizing epistemic malevolence as a type of deceptive behavior characterized by three features: (1) malevolence; (2) deception that leads to material harm; and (3) deceptive behavior that is grounded in a disposition.

### Epistemic Malevolence and Information use Environments

The literature on epistemic malevolence has mainly been concerned with describing what epistemic malevolence is, rather than with investigating the strategies organizations use to deceive. In particular, the existing empirical literature does not pay sufficient attention to the impact of an organization’s level of control over information. In this section, we introduce a distinction between two strategies of epistemically malevolent organizations which we will use to classify cases in our empirical analysis. Organizations ‘sow doubt’ when they contest information about them or their impacts that others control. By contrast, organizations ‘exploit trust’ when they deceive others by obfuscating, concealing, or falsifying information that they themselves control.

This distinction rests on Taylor’s ‘information use environment’ (Taylor, 1991). This is a foundational framework in the field of information science that shifts the locus of analysis away from the content to the context of purposive information use, focusing on the structure and situational dimensions of problems being worked on, the underlying assumptions of methods of problem resolution, and the physical and social settings in which information flows and use take place. The information use environment continues to be an active subject of theoretical inquiry and empirical investigation that has looked at the information use contexts and behaviors of managers, health care providers, lawyers, policy workers and other professions (Byström et al., 2019; Durrance et al., 2006; Jones, 2006; Olatokun & Ajagbe, 2010; Rosenbaum, 1993). Choo (2006, 2016) extended the use of the framework to examine the flow and use of information and their epistemic consequences in organizational settings.

We focus on the information use environment that is external to the organization, and comprises information flows and practices that concern a particular subject. We suggest that information use environments can be divided into ‘contested’ and ‘controlled’ environments—noting that this is a simplification, and that gradations between these two extreme types exist. In ‘contested’ information use environments, credible agents outside the organization have information about the organization or their impacts, e.g. independent researchers emphasizing the health risks of smoking. ‘Contested’ information use environments invite epistemically malicious organizations to ‘sow doubt’. For instance, tobacco companies misrepresented research findings, bribed researchers to publish amenable counter studies, and attempted to shift public debate away from their harmful behavior (Derry & Waikar, 2008; Michaels, 2008, 2020; Michaels & Monforton, 2005; Oreskes & Conway, 2011).

By contrast, organizations may find themselves in or even engineer an information use environment that is ‘controlled’. In controlled information use environments, external parties have no access to the relevant information about the organization or the organization’s impacts—the organization itself controls that information. ‘Controlled’ information use environments create opportunities for organizations to deceive by ‘exploiting trust’ others place in information provided by the organization.

Zaheer et al. (1998) and Harris and Zaheer (2006) have introduced the notion of inter-organizational trust to organization studies, and the concept has been used to study inter-organizational trust and distrust empirically (Oomsels & Bouckaert, 2014). Zaheer et al. define inter-organizational trust as the expectation that an actor can be relied on to fulfill obligations; will behave in a predictable manner; will act and negotiate fairly when the possibility for opportunism is present, and view betrayal as “an inherent feature of trust” (1998, p. 143). Unlike the strategy of ‘sowing doubt’, organizations ‘exploiting trust’ are not merely trying to contest claims by external parties in the public arena. Rather, they attempt to preempt researchers, regulators, journalists and NGOs from investigating the harms they cause in the first place. Hence, the strategy of ‘exploiting trust’ consists in using a ‘controlled’ information use environment to deceive, for instance by hiding or falsifying information.

## Methodology

In the preceding sections, we have conceptualized organizational epistemic malevolence as a type of organizational deception characterized by three features: (1) malevolence; (2) the deception leads the deceived to make decisions causing material harm; and (3) the deceptive behavior that is grounded in a disposition. Research on organizational deception has so far not explicitly studied epistemic malevolence. This gives rise to our first research question: *Is there evidence of epistemic malevolence in cases of organizational wrongdoing?* The answer matters for how useful the concept of epistemic malevolence is for understanding organizational wrongdoing.

Moreover, we have distinguished two information strategies epistemically malevolent organizations might use: ‘sowing doubt’ in ‘contested’ information use environments, and ‘exploiting trust’ in ‘controlled’ information use environments. This distinction matters practically because it has implications for which counter-measures are effective in identifying and addressing epistemic malevolence. In ‘contested’ information use environments, the challenge is to strengthen information providers that are independent of the relevant organizations. By contrast, in ‘controlled’ information use environments, regulators face the challenge of establishing that organizations engage in deception in the first place. This gives rise to our second research question: *In cases of organizational wrongdoing involving epistemic malevolence, is there evidence of organizations using the strategy of ‘exploiting trust’ rather than ‘sowing doubt’?*

### Data

We use the public Violation Tracker database, a database created by the non-governmental organization Good Jobs First (Mattera, 2019), that aggregates violations resolved by federal regulatory agencies and the Justice Department of the United States since the year 2000. We work with the dataset snapshot taken on 28 June 2021, which covers 490,309 cases from more than 300 different agencies with penalties of $669bn. Given its broad coverage, the database provides a detailed picture of corporate wrongdoing insofar as it has been identified and dealt with by US federal regulatory agencies and is often used in research on corporate crime (Raghunandan, 2021; Soltes, 2019). We analyzed cases based on the information source linked in the database. Information sources typically consist of consent orders, settlement agreements, and/or extended press releases by the federal agency that resolved the case. Most information sources contain multi-page descriptions of the case. Given the high stakes context of pending legal action, we can assume that the information provided in the information sources is factually accurate. However, the source materials by agencies we use is not compiled with the purpose of identifying epistemic vices, but with the goal of prosecuting organizational wrongdoing. Agencies are more likely to be successful in imposing payment of punitive damages on offending organizations if they can demonstrate that the wrongdoing was motivated. As a result, the dataset might introduce bias on two levels: First, agencies are more likely to pursue cases of motivated wrongdoing. Second, agencies are likely to be biased to identify motivation. Therefore, our sample is not suited for estimating the prevalence of epistemic malevolence.

We selected a sample of eighty high-penalty cases across the eight offense types that Violation Tracker database distinguishes. In each of the eight offense types, we selected the ten cases which attracted the highest penalties, for two reasons. First, we ensure that we cover cases that had a significant negative impact on stakeholders. Those eighty cases represent $236bn in penalties, or 35% of penalties imposed in the almost half a million cases in the database. Second, we wanted to cover cases that were well enough documented to answer our research questions. Our approach has the advantage of sampling across a variety of conditions of wrongdoing rather than single exemplars of epistemic vice (de Bruin, 2014). Details about the dataset and the selection process are available in the Online Supplemental Material.

### Classification Approach

Based on a review of the linked source material, we categorized each case to answer our research questions. Cases were categorized by two research assistants. The classification of each case was reviewed by the authors, and disagreements were resolved in discussion between research assistants and the authors.

Note that the sources we study have been compiled with the goal of prosecution, not investigation of the research questions we pursue in this paper. Hence, the information contained in the report, though often informative, often provides only limited insight in the questions we study. We address this issue by taking a conservative approach. We classify cases as involving epistemic malevolence only if we have textual evidence that our tests are met. Cases that do not meet the criteria are classified as containing insufficient evidence.

To investigate the first research question whether there is evidence of epistemic malevolence in cases of organizational wrongdoing, we categorized cases according to the following three questions:

Question 1: Does the case of organizational wrongdoing involve an act of epistemic malevolence?

To answer this question, we identify the adverse outcome. Behaviors are classified as epistemically malevolent if one of the harms that the offending organization causes is epistemic in nature. To classify the harm at issue as epistemic or non-epistemic, we focus on what the offending organization is accused of in the cases’ source material. We deem organizations to act epistemically malevolently when they malevolently deceive others, and if their deception consequently causes material harm, i.e. physical harm to people or environmental harm. Almost every act of organizational wrongdoing is accompanied by deception, as organizations seek to conceal the material harm they cause. By contrast, as discussed above, epistemic malevolence captures cases where others make harmful decisions *because* they have been deceived by the organization.

Question 2: What is the epistemic harm caused?

If the case involves an act of epistemic malevolence, we describe who is harmed and what the epistemic harm consists in. For instance, Merrill Lynch (Case 41) failed to disclose to investors key facts about the quality of billions of dollars of mortgage backed securities. This deception constitutes the epistemic harm caused by the organization. The deception also contributed to investors’ decision to buy the risky assets, resulting in billions of dollars in losses. These losses are the material harm investors suffered as a consequence of their own decision to buy the risky assets, against the backdrop of the deceptive behavior of the bank.

Question 3: Is the case of organizational wrongdoing rooted in the vice of epistemic malevolence?

For an act of deception to constitute epistemic malevolence, it needs to be rooted in a disposition, behavioral pattern, or attitude, rather than being merely an isolated behavior. Such features are not directly observable, but must be inferred. We suggest assessing whether organizations possess the vice of epistemic malevolence not based on a single criterion, but to rely on several markers, each of which provides prima facie evidence. This process is similar to how psychiatrists diagnose mental health disorders such as depression (Snaith et al., 1976).^3^ We propose the following three markers: (a) Does the case of organizational wrongdoing involve an act of epistemic malevolence? (Q1); (b) Is the case of organizational wrongdoing rooted in culture or governance?; (c) Is the organization a repeat offender regarding this misconduct category?

We consider only cases that involve an act of epistemic malevolence (Q1). We acknowledge that these criteria provide only indicative evidence for whether a behavior is grounded in epistemic vice. In particular, we do not include a criterion relating to whether governance structures support epistemic malevolence because our data does not allow us to assess the governance structures of offending organizations. Hence, we regard our criteria as a pragmatic attempt to proxy whether a behavior is likely rooted in epistemic vice. If the case meets neither criterion 2 nor 3, we attribute no evidence for the vice of epistemic malevolence; we attribute weak indicative evidence if the case meets one of the criteria; and strong indicative evidence if the case meets both criteria.

To operationalize criterion 2, whether a case of organizational wrongdoing is rooted in culture or governance, we ask whether the act that led to the adverse outcome executed over an extended period of time, and whether there were several actors involved in those behaviors. If the answer to both questions is yes, we classify wrongdoing as rooted in organizational culture or governance—and otherwise we classify as no evidence. To operationalize criterion 3, whether the organization is a repeat offender, we check whether the company has been penalized several times in the same primary misconduct category between 2000 and 2020 (the database contains 23 primary misconduct categories).

To investigate the second research question whether there is evidence of organizations using the strategy of ‘exploiting trust’ rather than ‘sowing doubt’, we categorized cases according to the following two questions:

Question 4: Is the strategy used one of ‘sowing doubt’ or of ‘exploiting trust’?

If the case involves epistemic malevolence, we classify cases as either pursuing the strategy of ‘sowing doubt’ or of ‘exploiting trust’. We answer that question by classifying the information use environment as ‘contested’ or ‘controlled’, depending on whether the deceived rely exclusively on information controlled by the deceiving organization, or whether the deceiving organization contests information controlled by independent others.

Question 5: Which Information behaviors are involved?

If the case involves epistemic malevolence, we describe the information behaviors evidenced in our sources. We distinguish between information obfuscation, information concealment, and information falsification (Buller et al., 1994). We attribute information behaviors to organizations when there is textual evidence of that behavior, assigning several information behaviors if the textual evidence warrants it (e.g. Case 69).

In the next section, we will report the findings organized according to our two research questions: First, we will draw our findings on questions 1–3 to make clear whether there is evidence of epistemic malevolence in cases of organizational wrongdoing. Second, we will address whether there is evidence of organizations using the strategy of ‘exploiting trust’ rather than ‘sowing doubt’, drawing on our findings regarding questions 4–5. The full case classifications are available in the Online Supplementary Material.

## Findings

Is there evidence of epistemic malevolence in cases of organizational wrongdoing?

Epistemic malevolence is involved in 60% of cases (*n =* 48). Note that this is not a good estimate of the prevalence of epistemic malevolence in general, because our sample is not random but focussed on high-penalty cases—see our discussion above. Yet despite the small number of cases that we study, the aggregate penalty amount of our sample represents more than a third of the total penalties inflicted by government agencies covered by the database. Therefore, it does allow for the conclusion that acts of epistemic malevolence play a notable role in organizational wrongdoing.

A clear example of a case that we classified as involving epistemic malevolence is from 2014, when the Consumer Financial Protection Bureau achieved a settlement with Merrill Lynch (now part of Bank of America) in a case of deceptive marketing (Case 41):

“Merrill Lynch regularly told investors the loans it was securitizing were made to borrowers who were likely and able to repay their debts. Merrill Lynch made these representations even though it knew, based on the due diligence it had performed on samples of the loans, that a significant number of those loans had material underwriting and compliance defects – including as many as 55 percent in a single pool” (US Department of Justice 2014b).

The harm that the bank stands accused of consists in deceiving customers, rather than using deception to cover up a separate material harm, hence making it a clear case of epistemically malevolent behavior. Based on deceptive marketing by the bank, customers made financial decisions that resulted in material losses. They bought risky financial products from Merrill Lynch that lost dramatically in value during the financial crisis of 2008/9, resulting in billions of dollars in cumulative losses.

By contrast, in line with our definition of epistemic malevolence, we have excluded other cases involving deception, where organizations use deception as a mere cover up of a pre-existing material harm. In these cases, deception does not result in further material harm, and hence these cases provide no evidence for epistemic malevolence. Consider the case of American Electric Power (Case 34). In 2017, the company agreed to a settlement of more than $4.6 billion because of past excess emissions. The material harm caused by the company is to emit pollutants exceeding permitted levels. The company attempted to deceive regulators about their past emissions to avoid penalties. Yet, their deception occurred only after the fact, and therefore did not play a causal role in the original organizational misconduct. This stands in contrast to the Merrill Lynch case, where the material harm occurred *because* customers had acted on the false information provided by the company. It is possible that the company’s deception caused material harm other than environmental harm. For instance, investors who invested in the company based on their falsely reported emissions might have been harmed in the sense that they made an investment decision that they otherwise would not have made. Yet in contrast to the Merrill Lynch case, this is purely speculative, we have no evidence for such harm.^4^

Some cases are complicated by the fact that they involve several stages, which need to be classified separately. For instance, the Justice Department settled a case with Toyota Motor Companies in 2014 for misleading consumers and the regulator about safety issues with a $1.2 billion financial penalty (Case 71). The underlying issue was that Toyota produced cars with sticky gas pedals, as well as gas pedals that could be trapped by the car’s floor mat. These issues led in some cases to unintended acceleration, causing deaths and injuries. This underlying security risk was neither intentionally fabricated nor is it epistemic in nature. However, the Justice Department’s sentence is focussed on a later stage in the process. It accuses Toyota of deception once the company had discovered the safety issues: “‘Rather than promptly disclosing and correcting safety issues about which they were aware, Toyota made misleading public statements to consumers and gave inaccurate facts to Members of Congress,’ said Attorney General Eric Holder.” (US Department of Justice 2014a). We classify the case as one of epistemic malevolence because the stage at issue in the settlement concerns harm that is epistemic in nature. The harm that Toyota directly caused is to deceive customers, resulting in further material harm. Had the company been upfront about its security issues, drivers could have taken precautions and deaths and injuries could have been avoided.

We find evidence of harm to customers’ and other stakeholders’ financial, educational, and physical well-being. As an example of harm relevant to physical wellbeing, consider that the pharma company GlaxoSmithKline participated in preparing, publishing and distributing a misleading medical journal article (Case 61). The article misreported that a clinical trial of Paxil demonstrated efficacy in the treatment of depression in underage patients, when the study failed to demonstrate efficacy. The epistemic harm caused is to deceive medical professionals and ultimately patients about the efficacy of the drug. As a result, doctors have made badly informed decisions about which drug to prescribe.

As an example of harm relevant to educational wellbeing, consider the case of Corinthian Colleges (Case 15). The education company claimed inflated placement rates in their advertisement. The epistemic harm is to mislead potential students about their prospects for working in their chosen field. As a result, students may have opted for doing a degree at a Corinthian College, when they would have been better served by a different college.

In all cases we analyzed, the organization can expect its deception to yield benefits. For instance, deceiving investors about the risks of mortgage backed securities predictably leads to higher sales of these products for the organizations. Epistemically malevolent organizations aim to undermine the knowledge of others only to the extent that it serves their own ends.

We find that in the vast majority of cases involving acts of epistemic malevolence, the behavior was rooted in culture, measured by whether the behavior was sustained by several groups in the organization over several years. Moreover, we find that virtually all of the organizations in our sample are repeat offenders. In 94% of cases (*n =* 45) categorized as acts of epistemic malevolence, both the culture and the repeat offender criterion are met. In addition, 3% of cases meet the repeat offender criterion only. We take this as strong prima facie evidence that in the majority of cases that we study, the offending organization’s act of epistemic malevolence is rooted in the vice of epistemic malevolence.

In cases of organizational wrongdoing involving epistemic malevolence, is there evidence of organizations using the strategy of ‘exploiting trust’ rather than ‘sowing doubt’?

We find that the ‘big tobacco playbook’, characterized by the strategy of ‘sowing doubt’, is well represented among the cases of epistemic malevolence we studied (19% of cases, *n =* 9). An example is the case of Purdue Pharma (Case 1). Purdue Pharma set up illegal kickback schemes and alleged educational activities, obfuscating information about the risks of opioid medication for patients. These activities deceived patients into accepting opioids marketed by the company as safe and effective treatments when they were shown to cause addiction, fuelling the opioid crisis in the United States. Given that there were independent scientific studies showing that opioids were addictive, Purdue Pharma operated in a ‘contested’ information use environment. As a result, it adopted the strategy of ‘sowing doubt.’

However, many more organizations in our sample pursue the goal of ‘exploiting trust’ (81% of cases, *n =* 39). Toyota (Case 71) provides an example of a company that attempted to ‘exploit trust’ in information it provided about the security of its vehicles. The company has misled consumers by concealing statements about safety issues that would cause its vehicles to accelerate unintentionally. For instance, the company suppressed internal evidence about safety issues that had been flagged and escalated by engineering teams. Once the first accidents had occurred, it misled customers to think the issue had been addressed by only recalling some of the models affected by the safety issues. The case of Volkswagen (Case 12) as well as of GlaxoKlineSmith (Case 61) described above are further examples of ‘exploiting trust.’

We found cases in 10 different industries for the strategy of ‘exploiting trust’, including financial services (*n =* 15), motor vehicles (*n =* 7), and pharmaceuticals (*n =* 7). Each of the information behaviors (concealment, falsification, obfuscation) are evident in our sample, with cases involving concealment (*n =* 36) more frequent than cases involving falsification (*n =* 19) or obfuscation (*n =* 12).

Sometimes, organizations even set up sophisticated systems that allow them to exercise exclusive control over the flow and interpretation of information, to establish the preconditions for ‘exploiting trust’. Consider Volkswagen’s emission scandal of 2020. Volkswagen aimed to hide the excessive amount of their vehicles' emissions by implanting defeat devices. As a result, regulators assessing the emissions under test conditions measured lower levels of emissions than the vehicles actually caused during normal use. By implanting the defeat devices, Volkswagen pre-empted the ability of regulators to identify the excessive emissions their vehicles caused. Hence, the company transformed the information use environment from a ‘contested’ into a ‘controlled’ environment. Originally, the information use environment was ‘contested,’ in that regulators had an independent source of information about the amount of emissions produced by the company’s vehicles. By implanting the defeat devices, the company created a ‘controlled’ information use environment, where regulators and customers were fully reliant on the company for information about vehicle emissions.

There are gradations of information use environments between the extremes of ‘controlled’ and ‘contested’ environments.^5^ In Volkswagen’s case, the company created a fully controlled information use environment only during laboratory emissions testing. Outside this setting, emissions could be measured accurately, which led to Volkswagen being caught. Information use environments can also be on the spectrum between ‘controlled’ and ‘contested’ if the information in question can be obtained from other sources than a single organization, but taking this route is costly—which is a situation regulators often find themselves in. In fact, we might conceive of ‘controlled’ environments as an extreme case of a ‘contested’ environment with one organization that acts as a dominant trusted provider of information. We have simplified the distinction for the purposes of our analysis, classifying cases as either involving ‘controlled’ or ‘contested’ information use environments based on whether the offending organization is sufficiently dominant as an information provider to be able to deceive outside stakeholders to cause significant epistemic harm.

The ways the cases of misconduct we studied were first discovered highlight the importance of devising new strategies to identify and address ‘epistemic malevolence’. In ‘contested’ information use environments that give rise to the strategy of ‘sowing doubt’, it is plain from the contradictory information shared by the contestants that at least one party is sharing false information. By contrast, in most cases of ‘exploiting trust’, the deceptive behavior went undetected for long periods of time. As long as an organization monopolizes potentially discrediting information, there are no obvious warning signals for regulators or others to follow up.

## Discussion

The lens of epistemic malevolence allows us to deepen our understanding of organizational wrongdoing in three ways: by emphasizing the extent to which organizations harm by deceit; by showing the extent to which an epistemically malevolent disposition is rooted in culture and governance; and by describing how the strategies of epistemically malevolent organizations are shaped by the extent to which they can control potentially compromising information about themselves.

60% of cases we studied involve epistemic malevolence (*n =* 48). It would be a mistake to discount such cases as ‘merely’ involving epistemic harm. We show that the epistemic harm that organizations cause leads, via the actions of the deceived parties, to material harm. For instance, Merrill Lynch deceived investors by failing to disclose to investors key facts about the quality of the loans underlying mortgage backed securities (Case 41). This epistemic harm contributed to investors’ decision to buy the risky assets, resulting in the material harm of billions of dollars in losses. Our case study shows that causing epistemic harm plays a major role in the highest-penalty cases of corporate wrongdoing in a wide range of industries.

From the perspective of virtue and vice epistemology, the important role of epistemic harm and the material harm caused in its wake comes as no surprise. This branch of epistemology emphasizes that each of us is radically dependent on testimony of others (Battaly, 2017; Fricker, 2007; Goldman & Whitcomb, 2011; Lackey & Sosa, 2006; Zagzebski, 1996). It is difficult to overstate how fundamental a departure this perspective is from the Cartesian hope that individuals can gain knowledge through introspection and observation. Rather than building our knowledge from scratch, we rely on others for anything from facts about what food is safe for us to eat to the size of the country we live in. Moreover, we increasingly rely on information about arcane aspects of the world to lead our lives, and much of that information is created by organizations. As a result, we increasingly put our trust in organizations, in the sense of the definition of inter-organizational trust by Zaheer et al. (1998) as the expectation that organizations can be relied upon to meet their obligations and act in good faith when the possibility of opportunism is present. Therefore, it matters to all of us that organizations create, share, and store information responsibly. The language of organizational epistemic virtue and vice capture the qualities and failings that make organizations (ir)responsible in dealing with information. Epistemic malevolence is a particularly harmful epistemic vice, as it consists in being motivated to harm others by deceiving them, i.e. act as the square opposite of a responsible testifier. Epistemically malevolent organizations merely create an appearance of trustworthiness to betray others. Because of their important role in creating and sharing information, organizations are in a position to inflict severe epistemic harm at scale.

Organizational vice epistemology theorizes that vices are dispositions, grounded in organizational culture and governance (Baird & Calvard, 2019; de Bruin, 2014; Dempsey, 2015). Our analysis supports an interactionist view between person and situation (Pervin, 1989). To the proponents of vice epistemology, we provide indicative evidence that the overwhelming majority of acts of epistemic malevolence are rooted in the vice of epistemic malevolence. All the organizations in our sample are repeat offenders, meaning that they have been penalized for wrongdoing in the same fine-grained category several times over a ten-year period. Moreover, in all but three of the 48 cases involving acts of epistemic malevolence, the behavior was sustained over time and by more than a few ‘bad apples’, often in several departments.

Yet, we also show that the environment matters greatly for how the vice of epistemic malevolence manifests itself. The existing literature has emphasized the strategy of ‘sowing doubt’, i.e. of contesting findings about harms caused by their products. This is the result of focusing on a narrow set of cases characterized by a ‘contested’ information use environment, such as tobacco companies contesting claims by researchers about the health risks associated with smoking. We show that a ‘controlled’ information use environment in which others rely on information provided by the offending organization leads to different characteristic behavior. The strategy of ‘exploiting trust’ consists in preempting that information about the harm organizations cause becoming publicly known in the first place.

Our analysis also makes a contribution to organization studies. Much research on organizational deception focuses on intra-organizational deception (Fleming & Zyglidopoulos, 2008; Hubbell, 2019; Jenkins & Delbridge, 2017) or on cases that primarily involve the strategy of ‘sowing doubt’ in ‘contested’ information use environments (Derry & Waikar, 2008; Michaels, 2008; Michaels & Monforton, 2005; Oreskes & Conway, 2011). The notion of epistemic malevolence highlights that some organizations may be disposed to epistemically malevolent behavior towards outside stakeholders due to their culture and governance. Using this new paradigm, we describe the deception strategy of ‘exploiting trust’, which has received little attention to date. Stepping back, the notion of epistemic vice offers a new paradigm which focuses on understanding knowledge acquisition and sharing, information systems and organizational learning as important determinants of the ethical behavior of organizations.

**Fig. 2:**
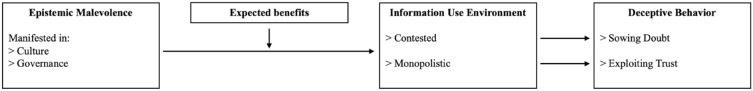
Model of pathways from epistemic malevolence to deceptive behavior

Figure 2 shows our model for how epistemic malevolence leads to organizational wrongdoing. The model shows a causal pathway from epistemic vice to malevolent acts of harm by deception. We suggest that some organizations have epistemically malevolent dispositions based on our indicative finding that acts of epistemic malevolence were overwhelmingly repeated, sustained over time, and involved more than a few individuals.

However, whether the vice of epistemic malevolence issues in acts of malevolent deception that constitute harm depends on two moderating factors. First, we suggest that organizations only engage in deception if the expected benefits to the organization or groups of individuals in the organization are sufficiently large. As we noted above, we have not encountered cases where organizations aimed to undermine the knowledge or understanding of others without expecting to gain.

Second, the deceptive behavior is moderated by the information use environment epistemically malevolent organizations either find themselves in or manage to create. Organizations in ‘contested’ information use environments tend to ‘sow doubt’, whereas organizations in ‘controlled’ information use environments ‘exploit trust’.

Understanding the importance of organizational epistemic malevolence and the associated deception strategies matters practically because we need to develop new capacities to identify and address this type of organizational wrongdoing. The question of how we can counteract epistemic vices with organizational epistemic virtues has not received much attention to date (Aikin & Clanton, 2010; Baehr, 2013). The key insight for legislators and regulators is that ‘exploiting trust’ calls for a different response than ‘sowing doubt’. To protect themselves from organizations ‘sowing doubt’, regulators can encourage independent research by cooperating with, for instance, academics, media, and NGOs. By contrast, when organizations exploit the trust placed in them as information providers, independent experts lack the data to identify the harmful impact to begin with. Hence, the primary bottleneck is to establish access to accurate information held by the epistemically malevolent organization.

Anecdotally, it seems the strategy of ‘exploiting trust’ may be on the rise as regulatory scrutiny shifts towards the tech sector. One of the allegations that whistleblower Frances Haughan made against Meta (then called Facebook) in October 2021 is that the company has hidden internal research showing that some of their services can be dangerous for children. Haughan alleges that the company chooses to ‘mislead and misdirect’ when it comes to its harmful impact on users (Stacey & Bradshaw, 2021). We mentioned Amazon’s attempt at privileging its own products in search results on their site in the introduction (Kalra & Stecklow, 2021). Meanwhile, Google has put in place sophisticated surveillance systems, seemingly designed to spot employees considering becoming whistleblowers (Krouse, 2021). What these cases have in common is that companies recognize that others are relying exclusively on them for information, or even create this reliance themselves. These developments call for regulators to take a more proactive approach in enforcing transparency standards and ensuring access to information monopolized by companies.

### Limitations and Future Research

We want to acknowledge three limitations. First, we studied high-penalty cases of organizational wrongdoing, using sources compiled by agencies with the goal of prosecuting organizations. It is possible that regulators frame behavior as intentional and attribute knowledge to the organization that it did not, in fact, possess. This would lead us to overestimate the importance of epistemic malevolence. Moreover, incomplete information may lead to bias about the presence of certain information behaviors, if, for instance, information falsification was more consistently reported than information obfuscation. Our conclusions about the mapping of information behaviors to information strategies should therefore be understood as tentative.

Second, the evidence on whether the organizations who commit acts of epistemic malevolence exhibit the vice of epistemic malevolence is merely indicative. We have suggested that to determine whether organizations possess the vice of epistemic malevolence, we should not rely on a single criterion but on several markers, each of which provides indicative evidence. We assess two criteria: whether the offending behavior is rooted in culture, and whether the organization is a repeat offender. A more detailed study of the cases we consider can improve our assessment method. There is, for instance, room for adding criteria for governance structures suggesting epistemic malevolence.

Our research suggests that there are features of an organization’s culture and governance that make it more or less prone to epistemically malevolent behavior. A question for further research is to what extent epistemically malevolent behavior is limited to high-penalty cases of wrongdoing, or to what extent it can be observed in more typical cases of organizational wrongdoing. If organizational malevolence is less prevalent in more typical samples, is there evidence of other epistemic vices?

We have only begun to map out information strategies and information behaviors associated with epistemic malevolence based on the information afforded by our sources. A next step would be to conduct more detailed case studies into exemplary cases to understand information strategies and behaviors in greater detail.

Finally, little work has been done on how to prevent and address epistemic malevolence in organizations. Much remains to be done in investigating the relationships between epistemic and material harms, crafting detailed interventions and regulatory strategies, as well as assessing their effectiveness.

## Conclusion

In connecting the discussion of epistemic malevolence to the empirical literature on organizational deception, we found a lack of attention to the impact of an organization’s ability to control compromising information on its deception strategy. We addressed this gap by conducting an empirical study of high-penalty organizational misconduct cases. We found that acts of epistemic malevolence are prevalent in high-penalty cases of organizational wrongdoing: There is evidence of epistemic malevolence in 60% of cases analyzed. Furthermore, we found indicative evidence that in the overwhelming majority of cases, organizational epistemic malevolence is rooted in epistemic vice. In the majority of cases involving epistemic malevolence, we found evidence of an underappreciated deception strategy: Rather than ‘sowing doubt’, organizations ‘exploit trust’ placed in them as information providers. This has important policy implications because the strategy of ‘exploiting trust’ calls for different counter-measures than the strategy of ‘sowing doubt’.

## Data Availability

https://osf.io/e2aqn/.

## Footnotes

1 We are grateful to Donald Palmer for pressing us on this point.

2 We are grateful to a reviewer for pushing us on the importance of multiple levels of analysis in organization studies.

3 We are grateful to Lisa Warenski for proposing this way of framing our methodology.

4 We are grateful to an anonymous reviewer for pressing us on this point.

5 We are grateful to an anonymous reviewer for pressing us on this point, and for suggesting the explanation of controlled information use environments as contested information use environments with a dominant trusted provider of information.
